# Diplosporous development in *Boehmeria tricuspis*: Insights from *de novo* transcriptome assembly and comprehensive expression profiling

**DOI:** 10.1038/srep46043

**Published:** 2017-04-06

**Authors:** Qing Tang, Gonggu Zang, Chaohua Cheng, Mingbao Luan, Zhigang Dai, Ying Xu, Zemao Yang, Lining Zhao, Jianguang Su

**Affiliations:** 1 Institute of Bast Fiber Crops, Chinese Academy of Agricultural Sciences, 348 West Xianjiahu Road, Changsha, Hunan, China

## Abstract

*Boehmeria tricuspis* includes sexually reproducing diploid and apomictic triploid individuals. Previously, we established that triploid *B. tricuspis* reproduces through obligate diplospory. To understand the molecular basis of apomictic development in *B. tricuspis*, we sequenced and compared transcriptomic profiles of the flowers of sexual and apomictic plants at four key developmental stages. A total of 283,341 unique transcripts were obtained from 1,463 million high-quality paired-end reads. In total, 18,899 unigenes were differentially expressed between the reproductive types at the four stages. By classifying the transcripts into gene ontology categories of differentially expressed genes, we showed that differential plant hormone signal transduction, cell cycle regulation, and transcription factor regulation are possibly involved in apomictic development and/or a polyploidization response in *B. tricuspis*. Furthermore, we suggest that specific gene families are possibly related to apomixis and might have important effects on diplosporous floral development. These results make a notable contribution to our understanding of the molecular basis of diplosporous development in *B. tricuspis.*

Apomixis is a naturally occurring mode of asexual reproduction in flowering plants. This process allows clonal reproduction through seeds, without meiosis and fertilisation, resulting in offspring that are genetically identical to the maternal plant^1^. The switch from a sexual pathway to an apomictic pathway entails at least three major steps: (1) circumvention of meiosis (a process called apomeiosis), (2) development of the embryo independently of fertilisation (i.e. parthenogenesis), and (3) formation of functional endosperm^2^. Although apomixis does not occur in major crop species, it is found in more than 400 species in 40 angiosperm families^3^,^4^. It attracts significant interest from an economic point of view, as the use of apomixis technology in major crop plants could potentially revolutionise agriculture. In addition, Sailer *et al*.^5^ showed that the phenotypes of *Hieracium pilosella* L. hybrids could be fixed across generations through apomixis, demonstrating that apomixis may potentially be used in plant breeding and agriculture.

Most apomicts are polyploid, and apomixis has previously been proposed to be a consequence of hybridisation and/or genome doubling, i.e. the events of polyploidisation^4^. However, polyploidisation alone is not sufficient to induce apomixis, as not all polyploids are apomicts^6^. In fact, through dosage regulation, polyploidisation leads to massive and widespread genomic changes, both in chromosome structure and on the level of gene regulatory networks, in sexual plants^7^,^8^,^9^,^10^,^11^. In addition, the switch from sexual to apomictic reproduction has also been associated with gene dosage effects during endosperm development^12^, and sequences with similar annotations have been reported to be associated with apomixis and/or polyploidy in various species, including *Poa pratensis*^13^, *Paspalum notatum*^14^,^15^, and *Eragrostis curvula*^16^,^17^. Therefore, for genotypes having different ploidy levels, a comprehensive analysis of gene expression alterations involved in apomixis and/or polyploidy response would be very valuable and provide the basis for future subselection of genes related to reproductive development.

Apomixis has clearly originated multiple times during the evolution of flowering plants, and therefore is polyphyletic in origin. Over the last two decades, several studies have demonstrated that apomixis is under genetic control^18^,^19^,^20^,^21^,^22^,^23^,^24^. Currently, there is some support for the hypothesis that apomixis is a consequence of deregulation of sexual reproduction^13^,^25^,^26^,^27^. Different experimental techniques such as map-based cloning approaches, differential display, subtractive hybridisation, and mimicking apomixis in sexual model plants have been used to investigate the genetic factors regulating apomixis and have led to the identification of many candidate genes^28^. These genes participate in many processes potentially central to apomixis, including protein degradation, transcription, stress response, and cell-to-cell signalling^14^,^27^,^29^,^30^,^31^. Recent research has highlighted the influence of plant hormones (such as auxin and cytokinin^32^,^33^,^34^) and epigenetic regulation^35^,^36^,^37^ on asexual embryogenesis, suggesting their involvement in apomixis induction and regulation. Conner *et al*.^38^ discovered that the *PsASGR-BBML* gene was expressed in egg cells before fertilisation and could induce parthenogenesis and produce haploid offspring in transgenic sexual pearl millet^38^. However, although considerable progress has been made in our understanding of apomixis in recent years, an understanding of its genetic control and molecular regulation remains very incomplete.

*Boehmeria tricuspis* (Hance) Makino has a basal chromosome number of x = 14, and whereas most individuals are triploid (2*n* = 42), diploid and tetraploid individuals have been found in nature. It is a hardy herbaceous or shrubby perennial, and diploid plants are wind pollinated, allogamous, and widely distributed along the Yangtze River in China. Plants are monoecious, with staminate axillary, paniculate inflorescences located below the pistillate inflorescences. It is related to *B. nivea* (L.) Gaudich., an important source of fibre (ramie); both species belong to the nettle family, Urticaceae. In a previous study, we confirmed that triploid *B. tricuspis* has obligate gynoecious apomixis, with the *Antennaria* type of diplospory, whereas diploid individuals appear to be monoecious and allogamous, with a *Polygonum*-type embryo sac^39^. Its life span, high seed yield, ease of cultivation, and obligate autonomous diplospory render triploid *B. tricuspis* an excellent plant for studying apomixis.

Next-generation sequencing (NGS) technologies are efficient, high-throughput, inexpensive, and reliable for transcriptome sequencing, and are suitable for non-model organisms such as *B. nivea*^40^. Recently, NGS has been employed to investigate changes in gene transcript abundance in apomicts relative to sexual individuals^41^,^42^, which has provided evidence for differential transcriptional pathways between them and enhanced our understanding of the complexity of gene expression, regulation, and networks in apomictic species. In the present study, we used the Illumina sequencing platform to focus on transcriptome differences between the global gene expression patterns of sexual and apomictic flowers over four developmental stages. These data provide a comprehensive resource for understanding apomictic development in *B. tricuspis* at the molecular level.

## Results

### Illumina sequencing and *de novo* assembly of the *B. tricuspis* transcriptome

**Figure 1:**
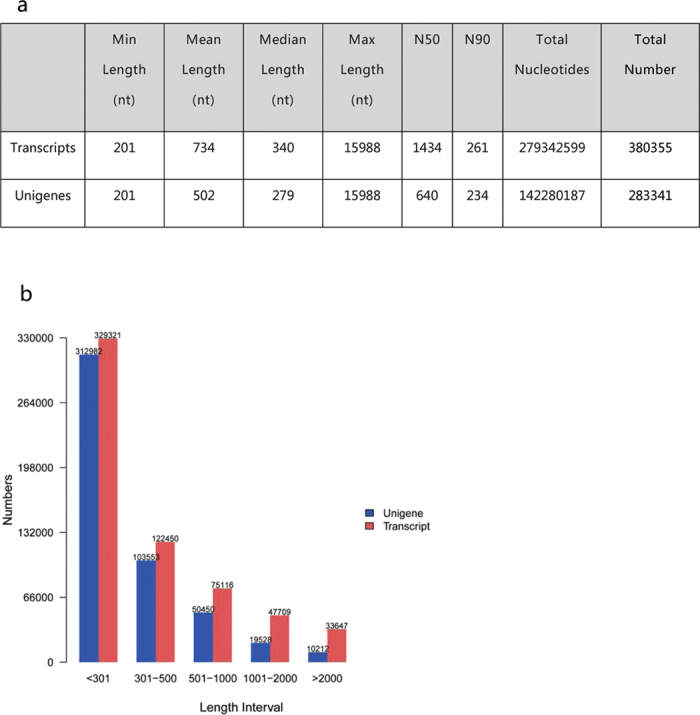
Statistics of *de novo* assembly of the transcriptome. (**a**) The statistics of *de novo* assembly of the transcriptome. (**b**) Distribution of the lengths of all transcripts and unigenes.

Flowers from an obligate apomictic triploid (A) and an obligate sexual diploid (S) were sampled at four developmental stages (Table 1): formation of the megaspore mother cell (MMC) (AI and SI), functional megaspores (AII and SII), mature embryo sac (AIII and SIII), and mature embryo (AIV and SIV). In total, 1,533 million short reads were generated from the 24 libraries, with 1,464 million clean reads selected for further analysis, and an average number of clean reads from each library of approximately 61 million. The G + C percentages were all approximately 48.25% (see Supplementary Table S1). The percentage of Q20 bases (those with a base Phred quality score >20 and an error rate <0.01) and Q30 (those with a base Phred quality score >30 and an error rate <0.01) were respectively higher than 97.17% and 93.51%, indicating the high quality of the raw sequencing reads of the samples (see Supplementary Table S1). After stringent quality assessment and data filtering with the Trinity *de novo* assembly programme, the sequences were assembled into a total of 380,355 transcripts (no less than 200 bp) with an N50 (length of the shortest transcript in the set of the longest transcripts comprising 50% of the genome) of 1,434 nt. The unigene dataset included 283,341 sequences with an N50 length of 640 nt and a mean length of 502 nt (Fig. 1a). The scatter plots of transcript and unigene size distributions are shown in Fig. 1b.

### Functional annotation of the transcriptome

The unigenes were annotated with the following databases: National Center for Biotechnology Information (NCBI) non-redundant protein (Nr, 178,606; 63.04%), NCBI non-redundant nucleotide sequence (Nt, 110,029; 38.83%), Kyoto Encyclopedia of Genes and Genomes (KEGG) Orthology (KO, 93,277; 32.92%), Swiss-Prot (173,923; 61.38%), Pfam (183,091; 64.62%), Gene Ontology (GO, 186,704; 65.89%), and Eukaryotic Orthologous Groups (KOG, 113,368; 40.01%). In total, 280,465 unigenes were found in at least one of these databases.

**Figure 2:**
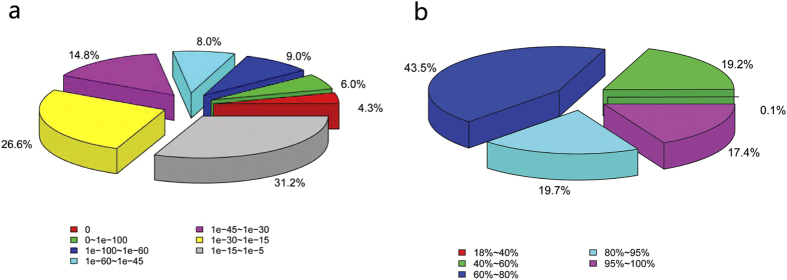
Results of a similarity search of the assembled sequences against the Nr database. (**a**) E value distribution of blast hits for each unique sequence with E value ≤10^−5^. (**b**) Similarity distribution of the top blast hits for each sequence.

**Figure 3:**
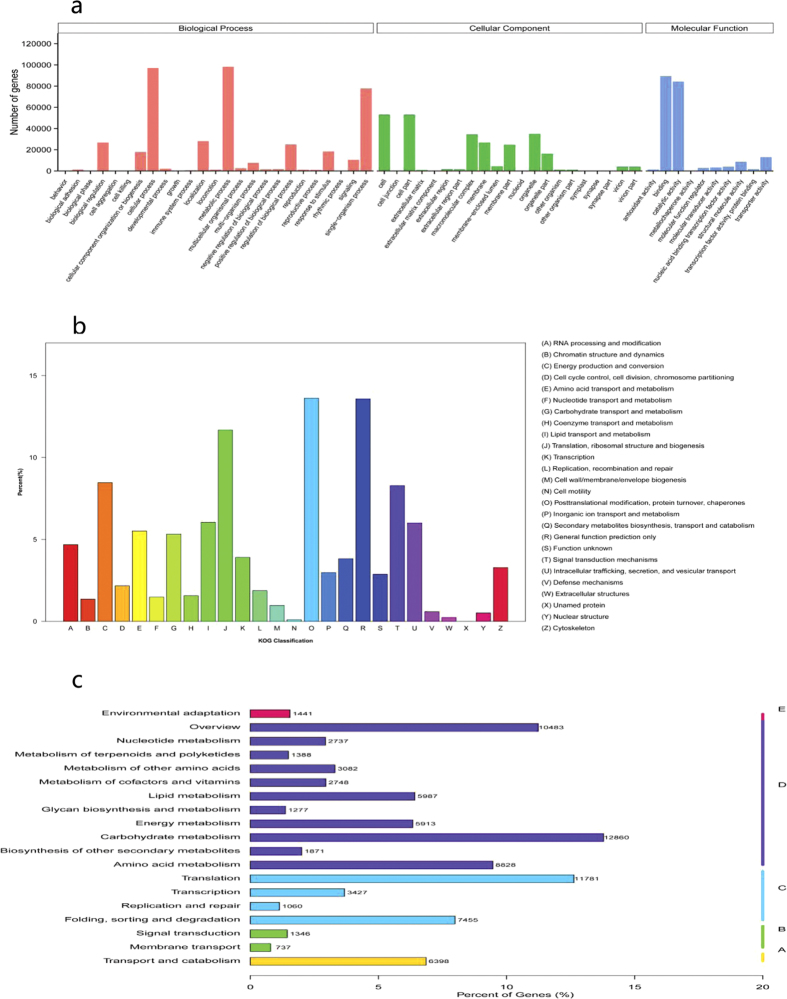
Analysis of functional categories of annotated unigenes. (**a**) Gene ontology classification of annotated unigenes. (**b**) KOG functional classification of all unigenes sequences. **(c)** KEGG classification of assembled unigenes. A, cellular processes; B, environmental information processing; C, genetic information processing; D, metabolism; E, organismal systems.

In the Nr annotation, 27.3% of sequences had perfect matches with E values <10^−45^, and 17.4% of the matches had a similarity over 95% (Fig. 2a,b). GO analysis was conducted on the 186,704 annotated unique sequences using the Blast2GO program. The annotated sequences were successfully assigned to GO categories of biological processes, cellular components, and molecular functions (Fig. 3a). The most enriched terms were metabolic processes (98,330), cellular processes (97,189), and single-organism processes (77,792) in the biological process category, and cells (53,137), cell parts (53,088), and organelles (34,918) in the cellular component category. Under molecular functions, binding (89,384) was most abundant, followed by catalytic activity (84,399) and transporter activity (13,080). The annotated unigenes were aligned to the KOG database for functional prediction and classification. In total, 113,368 sequences were assigned into 25 different KOG categories. The cluster for post-translational modification, protein turnover, and chaperones (13.63%) represented the largest group, followed by general function prediction only (13.58%), translation, ribosomal structure, and biogenesis (11.67%), energy production and conversion (8.47%), and signal transduction mechanism (8.29%) (Fig. 3b). KEGG pathway analyses provide information on the biological functions and interactions of genes. In total, 93,277 sequences were found to have significant matches in the database and were assigned to KEGG pathways (Fig. 3c). The pathways with the most representation among the unique sequences were carbohydrate metabolic pathways (12,860), followed by translation (11,781) and overview (10,483) pathways.

### Global analysis of differentially expressed genes

**Figure 4:**
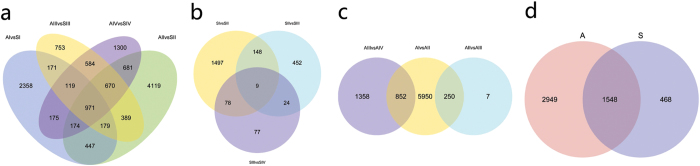
Venn diagrams showing overlap of differentially expressed genes (DEGs) between different developmental stages. (**a**) Venn diagram of DEGs between sexual and apomictic *Boehmeria tricuspis* at particular stages. (**b**) Venn diagram of DEGs in sexual *B. tricuspis* at four stages. (**c**) Venn diagram of DEGs in apomictic *B. tricuspis* at four stages. (**d**) Number of DEGs identified in sexual and apomictic *B. tricuspis*.

The normalised expression level (expected number of fragments per kilobase of transcript sequence per millions of base pairs sequenced, FPKMs) of each transcript was estimated in all the analysed samples. To characterise the developmental events of gene expression profiles, differences in gene expression at four stages during floral development in two genotypes were examined by comparing normalised expression values. Differentially expressed genes (DEGs) were identified by pairwise comparisons of different stages in the same genotype and of the same stages between different genotypes (Table 2). The number of DEGs detected in same-stage comparisons between the two genotypes was generally higher than that detected from same-genotype comparisons at different stages. Comparisons of different genotypes at the four stages identified 4,594 DEGs for AI vs. SI, 7,630 for AII vs. SII, 3,836 for AIII vs. SIII, and 4,674 for AIV vs. SIV. Additionally, different stages had specific DEGs: 2,358 in stage I, 4,119 in II, 753 in III, and 1,300 in IV (Fig. 4a). Comparisons of the four stages in S identified 1,732, 633, and 188 DEGs between SI and SII, SII and SIII, and SIII and SIV, respectively (Fig. 4b). Comparisons of the four stages in A identified 7,052, 257, and 2,210 DEGs between AI and AII, AII and AIII, and AIII and AIV, respectively (Fig. 4c). In total, 18,899 genes were differentially expressed in A and S at the four stages. Of these DEGs, 15,482 were differentially expressed in both genotypes during ovule development, whereas 2949 and 468 genes were differentially expressed in A and S, respectively (Fig. 4d).

**Figure 5:**
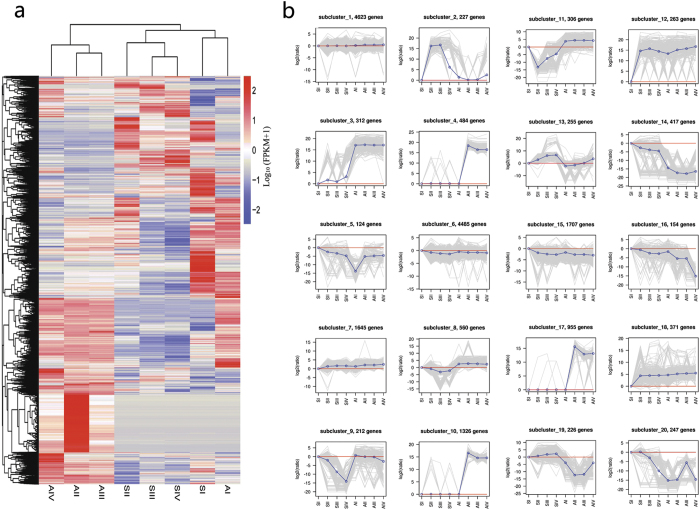
Clustering analysis of differentially expressed genes (DEGs). (**a**) Hierarchical clustering of DEGs. (**b**) Clustering of DEGs based on their expression patterns.

To further investigate gene expression profiles, we performed hierarchical clustering of all DEGs using the Euclidean distance method and complete linkage (Fig. 5a, b). Twenty clusters were plotted with distinctive expression patterns. Subclusters 1 and 6 containing 4,623 and 4,485 genes, respectively, possessed most of the DEGs, and the genes in the two clusters had similar expression patterns. Subclusters 4, 10, and 17 were characterised by peak expression in AII, and most of these genes were only expressed in A, indicating that the development stages were associated with specific gene clusters. Subcluster 13 included 255 genes that were progressively upregulated in both genotypes from stage I to IV, and the expression level of these genes in A was lower than in S. Most of the genes in subclusters 14, 15, and 16 were downregulated in both genotypes at later developmental stages, and the expression level in S was higher than in A.

### Validation of differential gene expression

We performed quantitative real-time PCR analysis to validate the results of differential gene expression obtained from the RNA-seq data. We observed similar gene expression trends (upregulation or downregulation) in RT-qPCR analysis as in RNA-seq for most of the samples. Further, we determined an overall correlation value of 0.78 (ranging from 0.59 to 0.93 for individual genes) between RNA-seq and qRT-PCR for all (total of 224; average fold change of 14 genes in the two genotypes at four developmental stages) the data points analysed (see Supplementary Fig. S1). These results indicated a very good agreement between the results obtained via RNA-seq and RT-qPCR.

### Functional classification of DEGs related to diplospory and/or polyploidy response

**Figure 6:**
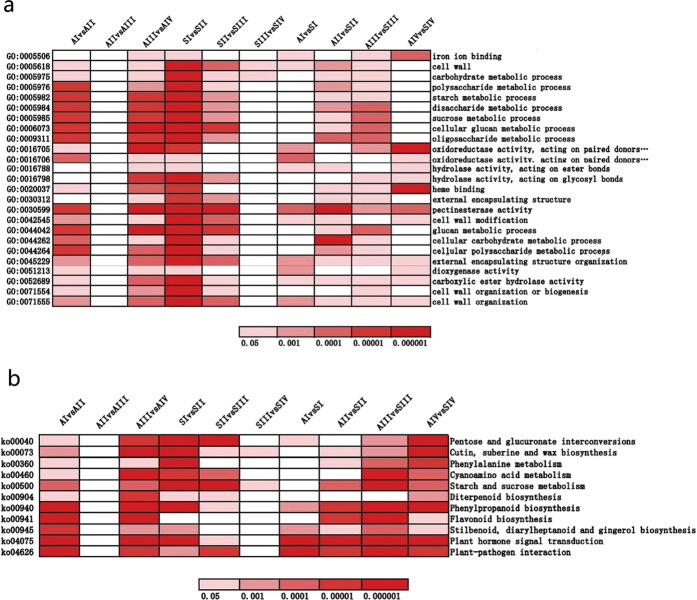
Heatmap showing expression profile of selected enriched GO terms and KEGG pathways of differentially expressed genes (DEGs). (**a**) The significances of the most represented GO-slims in each main comparison are indicated using log-transformed P-values (red). (**b**) The significances of the most represented KEGG pathways in each main comparison are indicated using log-transformed P-values (red).

We used GO assignments to classify DEG functions in pairwise comparisons of cDNA libraries between different genotypes and between different ovule developmental stages (see Supplementary Fig. S2). We selected 25 over-represented GO terms that were enriched in at least five comparisons to construct a heatmap showing expression profiles (Fig. 6a). No terms were significantly enriched in comparisons between AII and AIII, and only two were enriched for SIII vs. SIV. Genes in the categories of cell wall, cell wall organisation, external encapsulating structure organisation, carboxylic ester hydrolase activity, and pectinesterase activity were significantly enriched in eight pairwise comparisons. Four GO terms, pectinesterase activity, external encapsulating structure organisation, carboxylic ester hydrolase activity, and cell wall organisation, were significantly enriched in pairwise comparisons between the two genotypes at all four stages.

To further explore the biological pathways in which the DEGs are involved, we performed KEGG analyses (see Supplementary Fig. S3). Eleven over-represented KEGG pathways that were enriched in at least five pairwise comparisons were selected for a heatmap showing expression profiles (Fig. 6b). In these pathways, no term was greatly enriched in all comparisons. Five pathways, cutin, suberin, and wax biosynthesis, starch and sucrose metabolism, phenylpropanoid biosynthesis, plant hormone signal transduction, and plant–pathogen interaction, were significantly enriched in eight pairwise comparisons. Four pathways, phenylpropanoid biosynthesis; stilbenoid, diarylheptanoid, and gingerol biosynthesis; plant hormone signal transduction; and plant–pathogen interaction, were significantly enriched in pairwise comparisons between the two genotypes in all four stages, indicating that genes related to these processes play an important role in polyploidy response and/or diplosporous development in *B. tricuspis*.

### Genes related to polyploidy and/or diplospory

**Figure 7:**
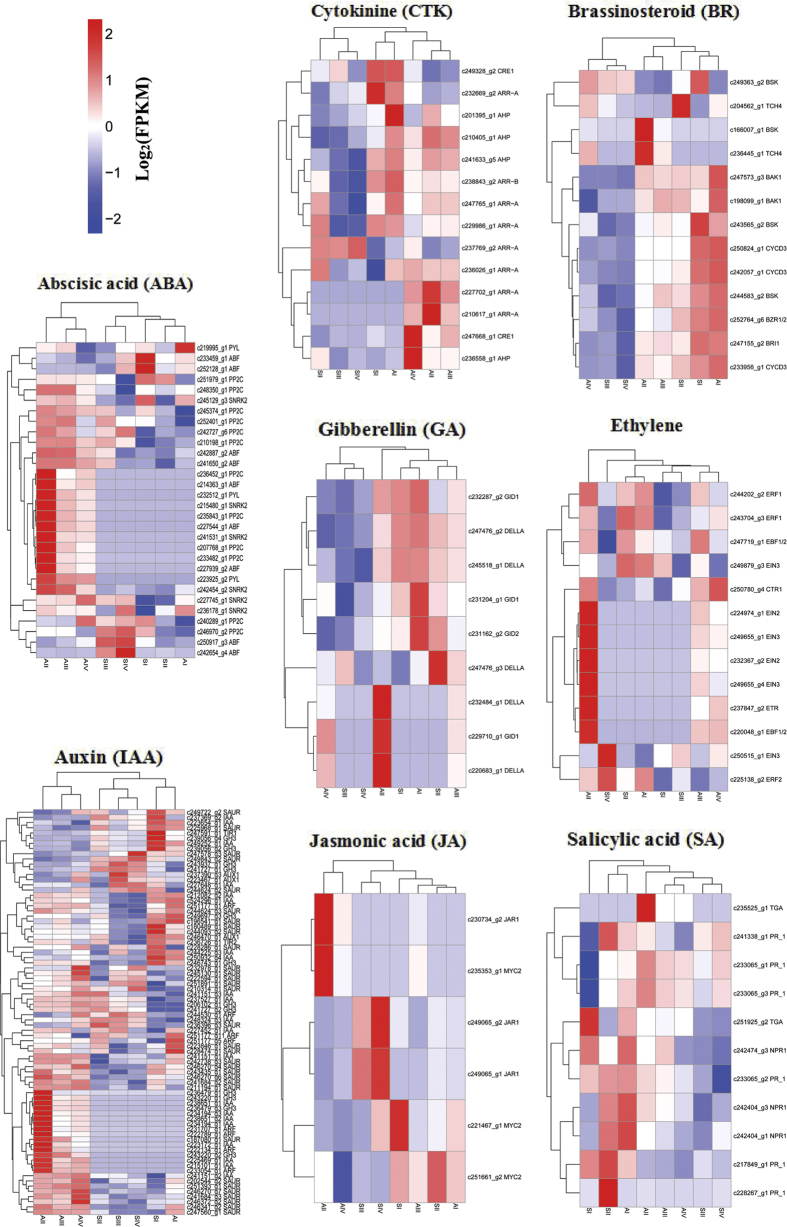
Heatmap of the expressed genes assigned to hormone signal transduction pathways. The log-transformed expression values range from −2 to 2. Red and blue indicate up- and down-regulated transcripts, respectively.

Plant hormone signal transduction pathways showed significant enrichment in pairwise comparisons between the two genotypes at all four stages, and given that auxin has previously been reported to be related to cell fate specification during embryo sac development^43^,^44^, we created a heatmap of the expressed genes assigned to hormone signal transduction pathways (Fig. 7). A fairly large number of DEGs assigned to hormone signal transduction, hormone-responsive proteins, hormone biosynthesis pathways, and hormone transporter proteins were identified. More DEGs are involved in the auxin (IAA) and abscisic acid (ABA) pathways than in the gibberellin (GA), cytokinin (CTK), ethylene, jasmonic acid (JA), salicylic acid (SA), and brassinosteroid (BR) pathways. The expression patterns of these genes were divided into many clusters, and gene homologues were distributed in different clusters in some cases. The gene expression patterns had higher similarity among developmental stages within each genotype than between genotypes. The results indicated that specific regulation of diplospory and/or polypoidy response might be governed by endogenous hormone levels.

**Figure 8:**
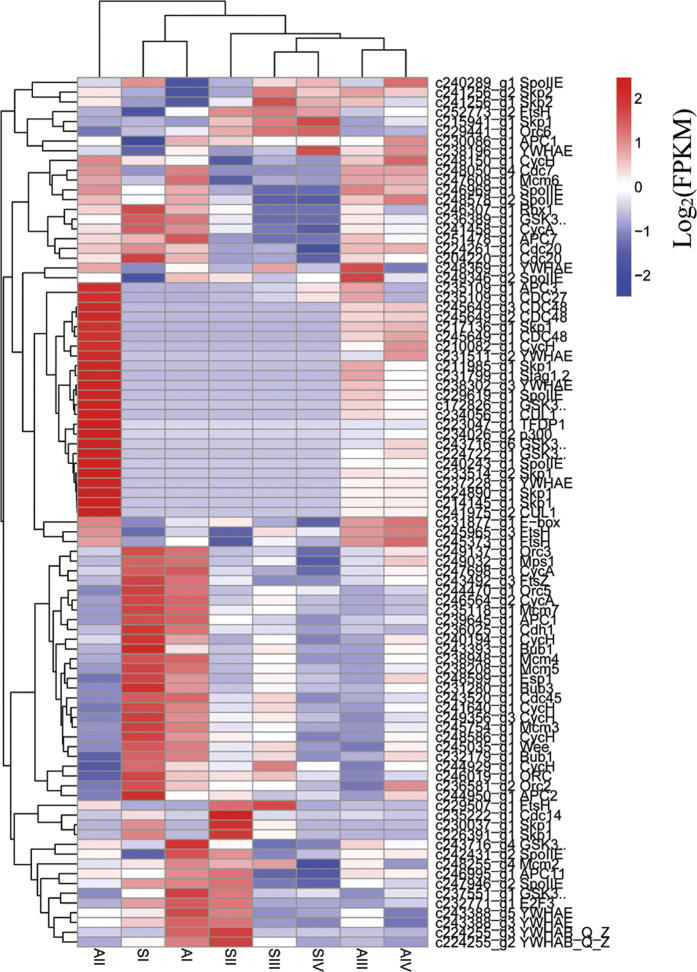
Heatmap of the expressed genes assigned to cell cycle pathways. The log-transformed expression values range from −2 to 2. Red and blue indicate up- and down-regulated transcripts, respectively.

In apomictic *B. tricuspis*, differentiated MMCs in ovules can bypass meiosis to produce unreduced megaspores that further divide mitotically to form an embryo sac. As cell cycle regulation is a crucial aspect of the apomictic pathway, we constructed a heatmap of 89 DEGs assigned to the cell cycle pathway (Fig. 8). This analysis revealed that gene expression profiles were most similar between adjacent developmental stages within each genotype. The AIII gene expression pattern was closest to that of AIV, and SIII was most similar to SIV. At stage I, the gene expression patterns between the genotypes were more similar. However, by stage II, gene expression patterns had diverged. The gene expression pattern of AII was the least correlated with that of the other samples, indicating specific regulation of the cell cycle process.

In-depth studies have revealed that transcription factors (TFs) are involved in plant reproductive regulation, particularly during embryo and endosperm development, and therefore we further analysed the TFs specifically expressed in sexual and apomictic *B. tricuspis*. Of these, 189 TFs belonging to 49 families were only expressed in apomictic *B. tricuspis* and 16 TFs in 11 families showed specificity in sexual *B. tricuspis* (see Supplementary Tables S2 and S3). Remarkably, in apomictic *B. tricuspis*, none of the specifically expressed TFs, except OFP, was expressed at AI, although these had expression peaks at AII. However, specifically expressed TFs in sexual *B. tricuspis* had different expression peaks. This indicated that diplosporous development, particularly at stage II (apomeiosis), is possibly associated with the functions of specific TFs.

## Discussion

On the basis of our DEG analysis, a total of 18,899 genes were identified that are significantly differentially expressed between the sexual and apomictic samples in at least one comparison. The number of DEGs identified between AII and SII was remarkably higher than that detected between AI and SI, AIII and SIII, or AIV and SIV, which had comparable numbers of DEGs (Fig. 4a). This indicates that the greatest difference in transcript representation between the two reproductive modes occurs during apomeiosis and meiosis. Similarly, the number of DEGs identified between AI and AII was also significantly higher than that detected between AII and AIII or AIII and AIV (Fig. 4c). This implies that the greatest gene expression change occurred at the apomeiosis stage during diplosporous development, which provides insights for further elucidating the regulatory mechanisms that control apomictic development. Among the identified DEGs, 15,482 (81.9%) were significantly regulated in both genotypes during floral development, and these genes appeared to be expressed at different times in the sexual and apomictic plants (Fig. 5b), which is consistent with the hypothesis that heterochronic changes in gene expression patterns are an underlying mechanism leading to apomixis from a sexual genetic background^26^. Of all the DEGs, 15.6% and 2.5% were differentially regulated in A and S, respectively, and 189 (6.4%) A-specific genes produced TFs belonging to 49 families, whereas only 16 (3.4%) S-specific genes produced TFs in 11 families. The number, percentage, and family richness of specific TFs were higher in apomictic regarding sexual samples, suggesting that a high number of molecular routes are being specifically modulated in diplosporous development.

Additionally, the total number of DEGs across the four stages was higher in A than in S, and there are almost 3000 transcripts expressed specifically in apomictic plants. Before we can postulate the existence of transcripts indicative of intricate molecular routes specifically activated during diplospory, which are repressed in sexual plants, we must take into consideration the fact that some of the variation observed in the apomictic triploids could be related to the higher number of alleles in these plants compared with diploids. Since the ratio of transcripts to unigenes was not provided for each library separately, we cannot compare the occurrence of transcript variants for each unigene in both sample types. Fortunately, 3000 transcripts is a sufficiently large number, because even if the apomictic triploids have 100% heterozygous loci there would be 1000 genes, which is double the number of S-specific DEGs. Accordingly, we can hypothesise that diplosporous development requires the activation of interrelated specific pathways involving a large number of genes, and that these genes are indicative of major reprogramming (mainly upregulation).

Plant hormones directly or indirectly regulate cell fate decisions and interact differentially in many developmental contexts^45^. In our KEGG analysis of DEGs, we found that plant hormone signal transduction pathways were not only significantly enriched in most within-genotype pairwise comparisons but also in pairwise comparisons between the sexual and apomictic genotypes (Fig. 6b) at all four stages, suggesting that alterations in plant hormone signalling might be important in apomictic development.

The phytohormone auxin affects cell division and elongation, differentiation, tropisms, apical dominance, senescence, abscission, and flowering, including embryogenesis and post-embryonic development^43^,^44^,^46^,^47^. Auxin might also affect apomictic ovule development^32^,^48^,^49^. In this study, we analysed DEGs involved in the auxin signal transduction pathway (Fig. 7) and found that three *AUX1* transcripts, two *TIR1* transcripts, 21 *IAA* transcripts, eight *ARF* transcripts, 12 *GH3* transcripts, and 32 *SAUR* transcripts were differentially expressed in pairwise comparisons of the samples. Seven *IAA* and four *ARF* transcripts were specifically expressed in apomictic *B. tricuspis* (see Supplementary Table S2). The expression patterns of these DEGs demonstrate that auxin signalling pathways are crucial and are dynamically regulated in apomictic and/or polyploidy development. IAA and ARFs are two large families of TFs that control the auxin transcriptional response. ARFs bind the promoters of auxin-responsive genes and either activate or inhibit transcription depending on the type of ARF^50^. The IAA proteins bind to the ARFs through shared domains, called domain III and IV in both proteins, and repress auxin-regulated transcription^51^. Rademacher *et al*.^52^ showed a pre-pattern of unique ARF combinations in the embryo that may act as an underlying template that defines different transcriptional cellular responses to auxin. Evidently, triploid and diploid *B. tricuspis* have distinct sets of ARFs in ovule development, which may result in a different reproductive output. Interestingly, specifically expressed ARFs and IAA were not expressed at AI and had expression peaks at AII, suggesting that auxin responses may affect fate decisions of differentiated MMCs, causing them to develop directly into a functional megaspore. Similarly, Polegri^53^ found that a homologue of *Arabidopsis* ARF1 was specifically expressed in the early stages of apomictic ovule formation in *Paspalum simplex* Morong, and suggested that auxin response may affect the differentiation of the aposporic initial from nucellus cells. We observed that a range of DEGs related to ABA, GA, CTK, ethylene, JA, SA, and BR were detected in our study (Fig. 7). These genes showed many different expression patterns, indicating that hormones, particularly IAA, play a vital role in apomictic and/or polyploidy development. Apomictic reproduction in *B. tricuspis* is a complex developmental process that might depend on the balanced expression of the genes related to hormone signal transduction pathways within a complex network.

Transcriptomic analysis of the developing flowers of diplosporous and sexual *B. tricuspis* identified 2,949 genes with A-specific expression. Among these, gene involved in the processes of chromatin structure modification, proteolysis, protein folding, carbohydrate, and signal transduction were detected, which may be regulated by ploidy changes^15^,^17^. Apart from those genes only controlled by ploidy, at least some of these are involved in diplospory in *B. tricuspis.* Because we have used full flowers, *in situ* hybridization techniques should be taken into account when validating the candidate genes related to diplosporous ovules development. On the basis of functional classification, we suggest that the following gene families are potentially valuable for future diplospory research.

The argonaute (AGO) gene family encodes four characteristic domains: N-terminal, PAZ, Mid, and a C-terminal PIWI domain. AGO proteins can use these functional domains to bind small non-coding RNAs and control protein synthesis, playing a central role in RNA silencing processes^54^. AGO proteins are involved in sRNA regulatory pathways in plants and have been associated with cell specification and embryo sac development^55^,^56^,^57^. In particular, the *ago 9* mutant in *Arabidopsis* exhibits apospory-like gametogenesis^56^, and the maize *ago 104* (the orthologue of *Arabidopsis ago 9*) mutant has diplospory-like gametogenesis^57^ In the present study, eight AGO transcripts were specifically expressed in apomictic ovules (see Supplementary Table S4). Moreover, the expression of these genes was the highest at stage AII. This suggests that these AGO transcripts are directly involved in apomeiosis in *B. tricuspis*.

Particular attention was paid to TFs specifically expressed in diplosporous *B. tricuspis.* The AP2-EREBP TF family was predominantly enriched, with 15 transcripts specifically expressed in diplosporous ovules (see Supplementary Table S2). The AP2-EREBP family contains the so-called AP2 DNA-binding domain, which has been identified in a wide range of plants. Within the family, there is a BBM-like subgroup that shares a conserved bbm-1 domain^58^. Ectopic expression of the *BBM* gene in *Arabidopsis* led to the formation of somatic embryos in seedlings^59^. Recently, a study revealed that the *PsASGR-BBML* gene, which is in perfect linkage with the *ASGR* of both *Pennisetum* and *Cenchrus*, could induce parthenogenesis or the initiation of embryo development from an unfertilised egg cell^38^. *ASGR-BBML* was strongly supported as a candidate gene for the apomictic function of parthenogenesis^60^. In contrast to their function at the stage of parthenogenesis in *Pennisetum, AP2* genes are expected to have important functions in diplosporous development in *B. tricuspis*.

Another likely family is the E3 ligases, which are part of the ubiquitin proteasome system and facilitate the transfer of ubiquitin moieties to a substrate protein, the preparative step for degradation via the 26S proteasome^61^. One peculiar important member of the E3s, SCF, dominates DNA duplication and cell division in all eukaryotes with the anaphase promoting complex/cyclosome (APC/C)^62^. SCF is a multimeric E3 composed of a CUL1, a RING-finger domain protein (RBX1), a ubiquitin-conjugating enzyme (E2), a specific substrate-recognition module composed of the adaptor Skp1, and one protein of the F-box family that physically interacts with the target substrate(s)^63^. We identified four *Skp1* transcripts and two *CUL1* transcripts there are specifically expressed in apomictic *B. tricuspis* (Fig. 8). Skp1 is a conserved subunit of SCF and is required for controlling the numbers of spores produced by a meiocyte in *Arabidopsis thaliana* (L.) Heynh^64^. In *Hypericum perforatum* L., an ARIADNE7-like E3 ligase (HpARI) was identified at the HAPPY locus and was proposed to act in a dominant negative manner at the protein level to influence alterations in gametophyte development^65^. Alteration in expression or function of the E3s, particularly of SCF, might therefore affect embryo sac development in *B. tricuspis*, leading to diplospory.

In summary, we sequenced flowers from a sexual and an obligate apomictic genotype of *B. tricuspis* at four key developmental stages using the Illumina platform. After comprehensive optimisation of *de novo* transcriptome assembly, a total of 283,341 unique transcripts were obtained from 1,464 million high-quality paired-end reads. Differential expression analysis revealed that differential plant hormone signal transduction, cell cycle regulation, and transcription factor regulation are possibly involved in apomictic development. In the future, we will use the list of candidates to select genes specifically related to diplospory by comparing apomictic and sexual genotypes of the same ploidy level. The present analysis contributes to the characterization of apomixis and provides valuable information on affected molecular pathways.

## Methods

### Plant materials and RNA extraction

Two *Boehmeria tricuspis* genotypes^39^, an obligate sexual diploid and an obligate apomictic triploid selected for the study were planted in the experimental field of the Institute of Bast Fiber Crops, Chinese Academy of Agricultural Sciences, Changsha, China. Flowers of the healthy female inflorescences at four development stages (Table 1) according to cytohistological investigations were collected and frozen immediately in liquid nitrogen and stored in −80 °C freezers before use. Three biological replicates were harvested from three individuals, and a total of twenty four samples were collected. Total RNA was extracted from flowers by using TRI Reagent (Sigma Life Science, USA), according to manufacturer’s instructions. The quality and quantity of RNA samples were assessed using NanoPhotometer^®^ Spectrophotometer (IMPLEN, CA, USA), Agilent Bioanalyzer 2100 (Agilent Technologies, CA, USA) and agarose gel electrophoresis. Only the RNA samples with 260/280 ratio between 1.8 to 2.0, 260/230 ratio between 2.0 to 2.5 and RIN (RNA integrity number) more than 8.0, were used for sequencing.

### Illumina sequencing and *de novo* assembly

Illumina sequencing was performed at Beijing Novogene Bioinformatics Technology Co., Ltd by using the Hiseq^TM^ 4000 platform according to the manufacturer’s instructions (Illumina, San Diego, CA). Briefly, a total amount of 1.5 μg RNA per sample was used as input material for the RNA sample preparations. Sequencing libraries were generated using NEBNext^®^ Ultra™ RNA Library Prep Kit for Illumina^®^ (NEB, USA) following manufacturer’s recommendations and index codes were added to attribute sequences to each sample. The clustering of the index-coded samples was performed on a cBot Cluster Generation System using TruSeq PE Cluster Kit v3-cBot-HS (Illumina) according to the manufacturer’s instructions. After cluster generation, the library preparations were sequenced on an Illumina Hiseq 4000 platform and 150 bp paired-end reads were generated. The raw-sequence reads data were deposited in NCBI Sequence Read Archive (SRA, http://www.ncbi.nlm.nih.gov/Traces/sra) with accession number SRP091360.

The raw sequence data of Fastq format were processed for various quality controls, including removal of low-quality reads (over 50% nucleotides had Phred^66^ quality scores <=20), reads containing primer/adaptor sequences and reads containing ambiguous bases (N > 10%). Ultimately, clean reads were obtained by this filtering process and assembled into unigenes with the Trinity^67^ (v20140413p1) with min_-_kmer_-_cov set to 2 by default and all other parameters set default.

### Functional annotation

All the unigenes were used for functional annotation by BLASTX (v2.2.28+) with an E-value cut-off of ≤10^−5^ against the NCBI Nr database, Nt database and Swiss-Prot database. Moreover, the assembled sequences were compared against Pfam database using HMMER (v3.0) with an Hmmscan e-value ≤0.01. CDS (coding sequence) was obtained by blasting against Nr databases and Swiss-Prot database, as well as by using ESTScan^68^. GO annotation for the unigenes was based on the best alignment of Nr and Pfam obtained using the Blast2GO (v2.5)^69^ program. The unigene sequences were also aligned to the Clusters of KOG database to predict and classify functions using BLASTX (v2.2.28+) with an E-value cut-off of ≤10^−3^. Pathway assignments were performed according to the KEGG pathway database using KAAS (KEGG Automatic Annotation Server) with E-value cut-off of ≤10^−10^. Identification of TF families in *B. tricuspis* transcriptome was carried out based on iTAK (v1.6) program (http://bioinfo.bti.cornell.edu/cgi-bin/itak/index.cgi).

### Differential expression analysis of unigenes

Gene expression levels were estimated by RSEM^70^ for each sample. The expression levels of unigenes were calculated using FPKMs to eliminate the effect of gene length and sequencing level on the calculation of gene expression. Differential expression analysis of two samples was performed using the DESeq R package (v1.10.1). DESeq provide statistical routines for determining differential expression in digital gene expression data using a model based on the negative binomial distribution. The resulting P values were adjusted using the Benjamini and Hochberg’s approach for controlling the false discovery rate. Genes with an adjusted P-value cut-off of <0.05 found by DESeq were assigned as differentially expressed. When DEGs were identified, functional enrichment analyses including GO and KEGG were performed to identify which DEGs were significantly enriched in GO terms or metabolic pathways. GO enrichment analysis of the DEGs was implemented by the GOseq R packages^71^ based Wallenius non-central hyper-geometric distribution, which can adjust for gene length bias in DEGs. GO terms with corrected P value less than 0.05 were considered significantly enriched by differential expressed genes. KOBAS (v2.0)^72^ software was used to test the statistical enrichment of DEGs in KEGG pathways. The pathways with corrected P value less than 0.05 were defined as those with genes that display significant levels of differential expression.

### Clustering analysis

K-Means clustering was performed by Euclidean distance method and each centroid was the mean of the points in that cluster. Hierarchical clustering of gene expression was performed by clustergram function in Matlab Bioinformatics toolbox with default settings.

### Real-time PCR analysis

14 DEGs were randomly selected for validation using quantitative real-time PCR. Primers for real-time PCR, which were designed with the Primer3, (v.4.0.0) software, are listed in Supplementary Table S5. For each sample, first-strand cDNAs were reverse-transcribed from RNAs treated with DNase I (Fermentas, Canada) using M-MuLV Reverse Transcriptase (Fermentas, Canada) according to the manufacturer’s instructions. RT-qPCR was performed using an optical 96-well plate with an iQ5 multicolor real time PCR system (Bio-RAD, USA). Each reaction contained 1.0 μL of cDNA template from the reverse-transcribed reaction mentioned above, 10-nM gene-specific primers, 10 μL of iTaq™ Universal SYBR Green supermix (Bio-RAD, USA) in a final volume of 20 μL. The *EF1α* gene was selected for the internal control^73^. The thermal cycle used was as follows: 95 °C for 3 min, followed by 40 cycles of 95 °C for 15 s, 55 °C for 30 s. The real-time PCR analysis was performed with three biological replicates for each sample and three technical replicates of each biological replicate. The transcript level of each gene was normalized and fold change was calculated using standard 2^−ΔΔCT^ method.

## Additional Information

**How to cite this article**: Tang, Q. *et al*. Diplosporous development in *Boehmeria tricuspis*: Insights from *de novo* transcriptome assembly and comprehensive expression profiling. *Sci. Rep.* **7**, 46043; doi: 10.1038/srep46043 (2017).

**Publisher's note:** Springer Nature remains neutral with regard to jurisdictional claims in published maps and institutional affiliations.

## Supplementary Material

Supplementary Information

## Figures and Tables

**Table 1:** Stages of flower development in sexual and apomictic *Boehmeria tricuspis*.

Sample	Reproduction	Ovule Stage^a^	Development
AI	Apomixis	2A-B	MMC formed
AII	Apomixis	2C-E	Functional megaspores formed (apomeiosis)
AIII	Apomixis	2F-M	Mature embryo sac formation (megagametogenesis)
AIV	Apomixis	2Q-T	Mature embryo formation (autonomous endosperm)
SI	Sex	3A-B	MMC formed
SII	Sex	3C-I	Functional megaspores formed (tetrad to degeneration of megaspores)
SIII	Sex	3J-M	Mature embryo sac formation
SIV	Sex	3Q-T	Mature embryo formation

^a^According to Q.T. *et al*. (2016).

**Table 2:** Number of differentially expressed genes of pairwise comparisons used to identify the differentially expressed unigenes.

	SI	SII	SIII	SIV	AI	AII	AIII	AIV
SI		1732	2632	3938	4594			
SII	1732		633	1786		7630		
SIII	2632	633		188			3836	
SIV	3938	1786	188					4674
AI	4594					7052	3448	7609
AII		7630			7052		257	3023
AIII			3836		3448	257		2210
AIV				4674	7609	3023	2210	
