# Supplementary material for: Diplosporous development in Boehmeria tricuspis: Insights from de novo transcriptome assembly and comprehensive expression profiling
Source: Sci Rep. 2017 Apr 6;7:46043. doi: 10.1038/srep46043 (PMC5382578; doi:10.1038/srep46043)
Supplement: Supplementary Information [file srep46043-s1.pdf]

Apomictic development in *Boehmeria tricuspis*: Insights from *de novo* transcriptome assembly and comprehensive expression profiling

Qing Tang[1], Gonggu Zang[1], Chaohua Cheng[1], Mingbao Luan[1], Zhigang Dai[1], Ying Xu[1], Zemao Yang[1], Lining Zhao[1*] & Jianguang Su[1*]

Authors' addressesses:

[1]Institute of Bast Fiber Crops, Chinese Academy of Agricultural Sciences, 348 West Xianjiahu Road, Changsha, Hunan, China.

***Corresponding authors: E-mail:** csbzln@163.com or jgsu2016@163.com

Tel/Fax: +86-731-88998539

List of Supplementary Materials

Figure S1. Quantitative real-time PCR analysis to validate the results of RNA-seq.

Figure S2. GO terms significantly enriched in differentially expressed genes in comparisons of AI, AII, AIII, AVI, SI, SII, SIII, and SIV libraries.

Figure S3. KEGG pathways significantly enriched in differentially expressed genes in comparisons of AI, AII, AIII, AVI, SI, SII, SIII and SIV libraries.

Table S1. Summary of the read data generated.

Table S2. The expression of specific transcription factors in apomictic *Boehmeria tricuspis*.

Table S3. The expression of specific transcription factors in sexual *Boehmeria tricuspis*.

Table S4. The expression of specific *AGO* transcripts in apomictic *Boehmeria tricuspis*.

Table S5. List of primers sequences used for real time PCR analysis.

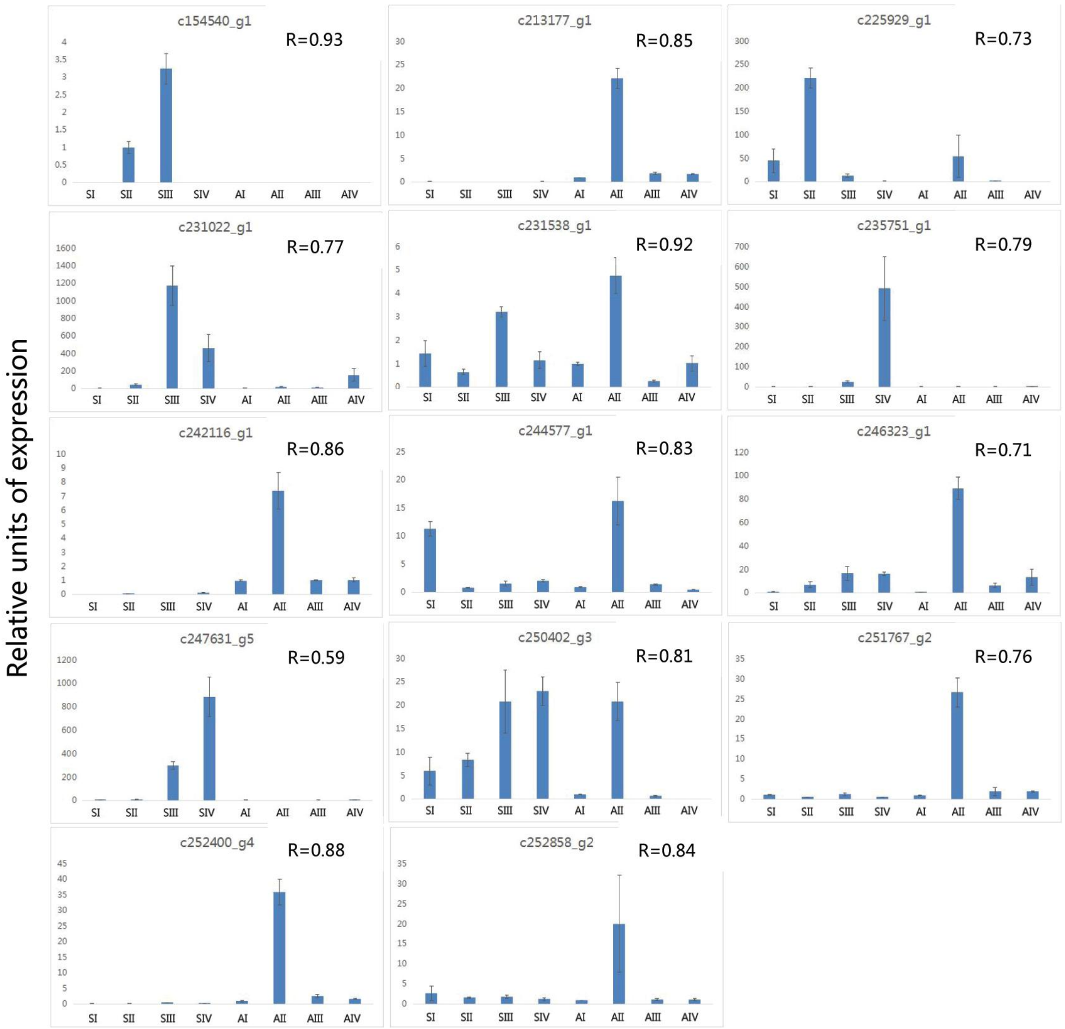

Figure S1. Quantitative real-time PCR analysis to validate the results of RNA-seq. The bar graph shows relative units of expression of differentially expressed genes obtained via RT-qPCR and RNA-seq analyses. 'R' represents correlation coefficient between RNA-seq and RT-qPCR data.

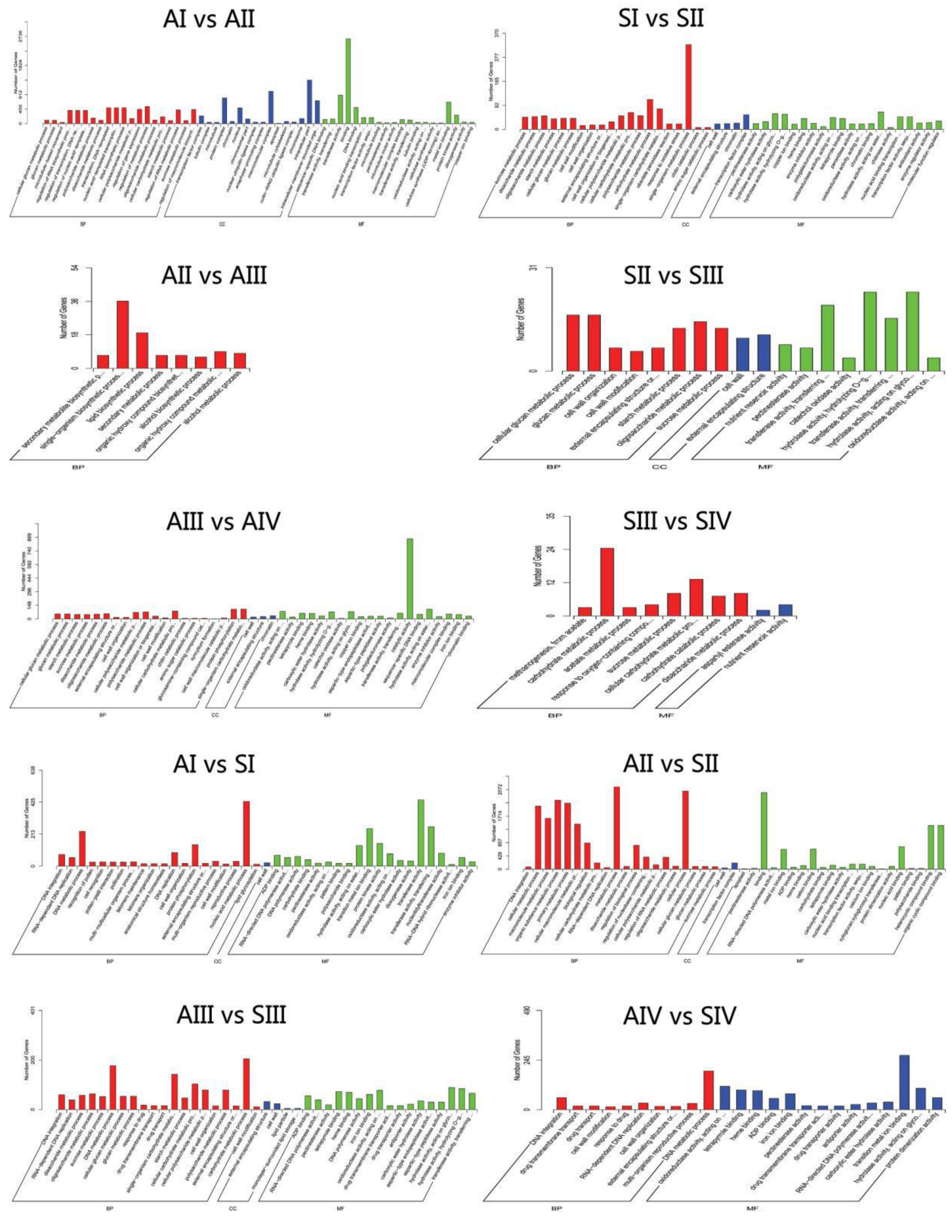

Figure S2. GO terms significantly enriched in differentially expressed genes in comparisons of AI, AII, AIII, AVI, SI, SII, SIII, and SIV libraries.

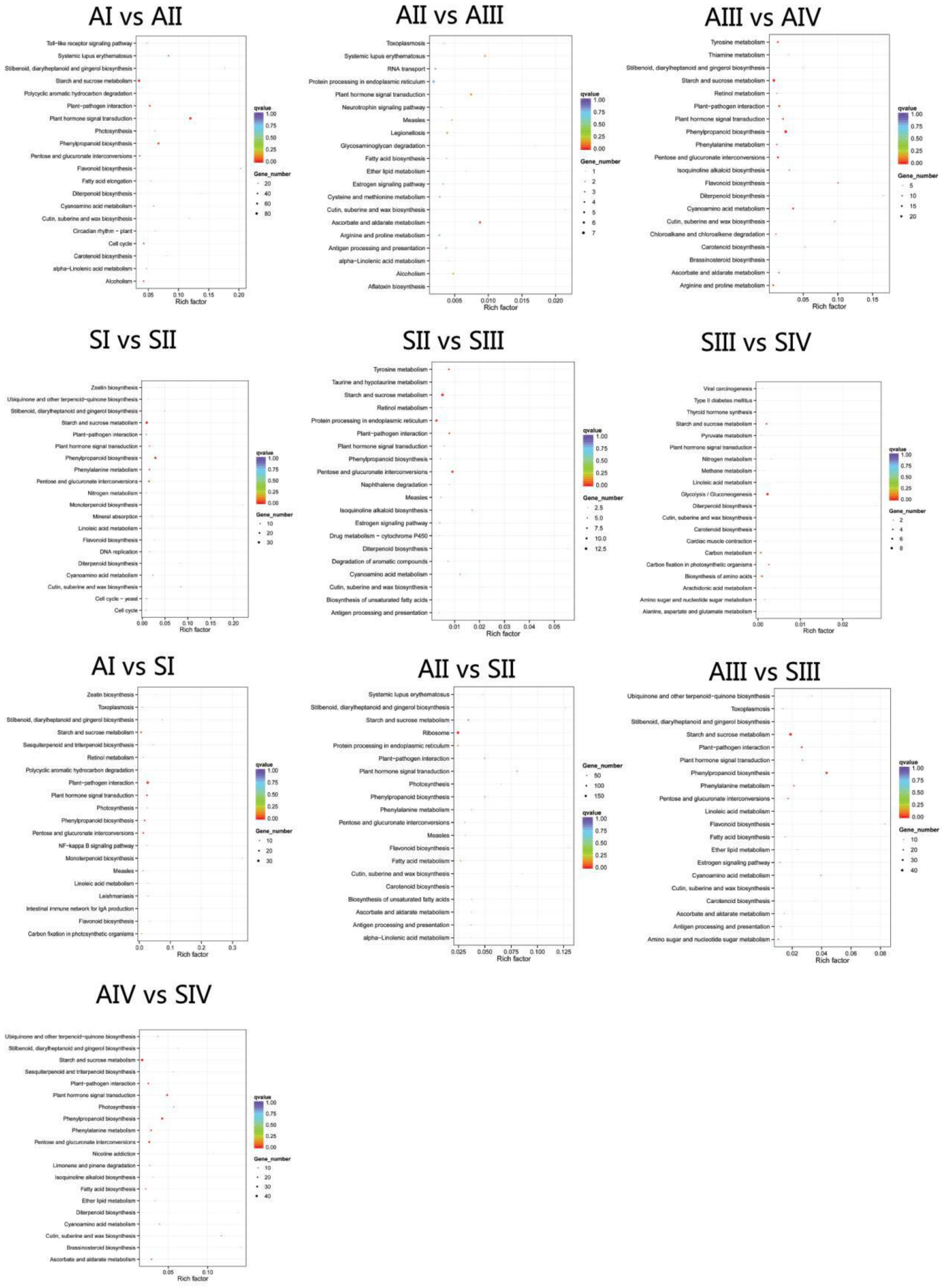

Figure S3. KEGG pathways significantly enriched in differentially expressed genes in comparisons of AI, AII, AIII, AVI, SI, SII, SIII and SIV libraries.

Sample	Raw Reads	Clean Reads	Clean Bases	Error (%)	Q20 (%)	Q30 (%)	GC (%)
SI_1	65790466	63321108	9.5G	0.01	97.40	94.02	46.50
SI_2	66653202	64290716	9.64G	0.01	97.60	94.41	47.85
SI_3	67215780	64670356	9.7G	0.01	97.31	93.79	48.12
SII_1	55301966	52842198	7.93G	0.02	95.95	90.79	48.99
SII_2	54308878	51918976	7.79G	0.02	95.91	90.75	47.80
SII_3	55703470	52961844	7.94G	0.02	95.90	90.65	49.79
SIII_1	51048888	48811342	7.32G	0.02	95.88	90.58	49.31
SIII_2	58350450	55742656	8.36G	0.02	96.22	91.25	48.90
SIII_3	55450906	52641306	7.9G	0.02	95.71	90.28	49.37
SIV_1	69464860	66595504	9.99G	0.01	97.37	94.04	49.98
SIV_2	59538072	56315972	8.45G	0.01	97.35	93.90	50.24
SIV_3	61279318	58870186	8.83G	0.01	97.38	93.98	50.64
AI_1	63455718	61291326	9.19G	0.01	97.59	94.37	47.72
AI_2	68547988	65700076	9.86G	0.01	97.26	93.79	49.08
AI_3	77232116	74487574	11.17G	0.01	97.59	94.43	47.40
AII_1	75435986	71976286	10.8G	0.01	97.75	94.81	47.55
AII_2	75887178	72323178	10.85G	0.01	97.69	94.74	47.46
AII_3	66660460	63679434	9.55G	0.01	97.72	94.75	47.58
AIII_1	62284260	58816380	8.82G	0.01	97.79	94.96	48.21
AIII_2	59886736	56683226	8.5G	0.01	97.73	94.78	47.52
AIII_3	60095700	56819754	8.52G	0.01	97.76	94.87	47.80
AIV_1	77336788	73351942	11G	0.01	97.74	94.79	46.68
AIV_2	60630894	57392718	8.61G	0.01	97.70	94.72	47.30

AIV_3	65572856	62114144	9.32G	0.01	97.79	94.86	46.29
All	1533132936	1463618202	219.54G				
Average	63880539	60984091	9.15G	0.01	97.17	93.51	48.25

Table S1. Summary of the read data generated.

Gene ID	Family	FPKM value							
		AI	AII	AIII	AIV	SI	SII	SIII	SIV
c223559_g1_1	ABI3VP1	0	0.74	0.12	0.14	0	0	0	0
c241015_g2_3	ABI3VP1	0	0.70	0.15	0.18	0	0	0	0
c228239_g1_0	ABI3VP1	0	0.60	0.13	0.14	0	0	0	0
c240284_g2_1	Alfin-like	0	2.00	0.39	0.21	0	0	0	0
c220290_g1_1	Alfin-like	0	1.01	0.24	0.17	0	0	0	0
c222549_g1_1	AP2-EREBP	0	1.57	0.33	0.48	0	0	0	0
c232292_g3_2	AP2-EREBP	0	1.38	0.31	0.32	0	0	0	0
c233140_g1_5	AP2-EREBP	0	0.95	0.35	0.26	0	0	0	0
c226181_g1_5	AP2-EREBP	0	0.93	0.33	0.46	0	0	0	0
c245619_g1_5	AP2-EREBP	0	3.11	0.97	0.83	0	0	0	0
c245619_g2_3	AP2-EREBP	0	2.28	0.39	0.49	0	0	0	0
c232447_g1_0	AP2-EREBP	0	0.67	0.15	0.22	0	0	0	0
c235724_g1_0	AP2-EREBP	0	0.92	0.14	0.19	0	0	0	0
c227256_g1_5	AP2-EREBP	0	0.73	0.22	0.23	0	0	0	0
c234378_g1_5	AP2-EREBP	0	1.74	0.28	0.36	0	0	0	0
c211923_g1_1	AP2-EREBP	0	1.08	0.30	0.00	0	0	0	0
c237924_g1_4	AP2-EREBP	0	0.81	0.22	0.30	0	0	0	0

c237924_g2_3	AP2-EREBP	0	0.75	0.22	0.12	0	0	0	0
c9219_g1_3	AP2-EREBP	0	1.06	0.32	0.47	0	0	0	0
c229098_g1_5	AP2-EREBP	0	1.16	0.35	0.33	0	0	0	0
c233054_g1-1	ARF	0	0.35	0.16	0.17	0	0	0	0
c231707_g1_1	ARF	0	0.39	0.04	0.11	0	0	0	0
c222789_g1-2	ARF	0	0.41	0.06	0.07	0	0	0	0
c222134_g1_2	ARF	0	0.41	0.15	0.04	0	0	0	0
c242510_g1_5	ARID	0	0.76	0.30	0.18	0	0	0	0
c232296_g1_2	ARID	0	0.43	0.15	0.07	0	0	0	0
c238651_g2_3	AUX/IAA	0	0.67	0.12	0.18	0	0	0	0
c225469_g1_4	AUX/IAA	0	0.67	0.22	0.33	0	0	0	0
c234194_g3_3	AUX/IAA	0	2.39	0.34	0.41	0	0	0	0
c215101_g1_1	AUX/IAA	0	0.51	0.19	0.24	0	0	0	0
c223172_g1_4	AUX/IAA	0	0.55	0.16	0.09	0	0	0	0
c234194_g1_4	AUX/IAA	0	1.74	0.23	0.33	0	0	0	0
c238651_g1_5	AUX/IAA	0	0.37	0.11	0.12	0	0	0	0
c233860_g3_2	BBR/BPC	0	0.56	0.08	0.15	0	0	0	0
c235353_g1_1	bHLH	0	0.91	0.15	0.09	0	0	0	0
c250413_g1_0	bHLH	0	6.51	2.91	0.80	0	0	0	0
c227747_g2_3	bHLH	0	0.45	0.19	0.10	0	0	0	0
c237856_g1_1	bHLH	0	0.72	0.25	0.21	0	0	0	0
c236415_g1_5	bHLH	0	0.45	0.29	0.29	0	0	0	0
c238243_g1_3	bHLH	0	1.29	0.37	0.29	0	0	0	0
c229179_g1_0	bHLH	0	0.77	0.66	0.08	0	0	0	0

c247767_g1_2	bHLH	0	13.71	4.11	4.62	0	0	0	0
c207919_g1_0	BSD	0	0.55	0.16	0.28	0	0	0	0
c228027_g1_1	bZIP	0	0.55	0.05	0.14	0	0	0	0
c229520_g1_4	bZIP	0	0.78	0.17	0.22	0	0	0	0
c233462_g3_5	bZIP	0	0.86	0.15	0.16	0	0	0	0
c227544_g1_1	bZIP	0	0.42	0.16	0.13	0	0	0	0
c227939_g2_1	bZIP	0	0.68	0.19	0.13	0	0	0	0
c233462_g1_4	bZIP	0	0.62	0.21	0.16	0	0	0	0
c245262_g3_1	bZIP	0	0.44	0.12	0.14	0	0	0	0
c214363_g1_5	bZIP	0	0.61	0.14	0.19	0	0	0	0
c222289_g1_1	bZIP	0	0.47	0.08	0.11	0	0	0	0
c221148_g1_5	bZIP	0	0.42	0.02	0.07	0	0	0	0
c229458_g1_4	bZIP	0	0.86	0.12	0.11	0	0	0	0
c235525_g1_3	bZIP	0	0.81	0.12	0.11	0	0	0	0
c236479_g2_1	C2C2-Dof	0	1.12	0.10	0.17	0	0	0	0
c249832_g5_4	C2C2-GATA	0	0.67	0.05	0.34	0	0	0	0
c209068_g1_4	C2C2-GATA	0	0.57	0.09	0.17	0	0	0	0
c232513_g1_3	C2H2	0	0.61	0.24	0.44	0	0	0	0
c244485_g3_1	C2H2	0	0.57	0.15	0.05	0	0	0	0
c225994_g1_5	C2H2	0	1.05	0.17	0.35	0	0	0	0
c237159_g1_0	C2H2	0	1.11	0.25	0.21	0	0	0	0
c19342_g1_3	C2H2	0	0.49	0.10	0.16	0	0	0	0
c237220_g1_4	C2H2	0	0.82	0.28	0.32	0	0	0	0
c209725_g1_0	C2H2	0	0.54	0.15	0.21	0	0	0	0

c224075_g1_1	C2H2	0	0.05	0.06	0.56	0	0	0	0
c225750_g1_5	C2H2	0	1.41	0.08	0.07	0	0	0	0
c237220_g2_4	C2H2	0	0.66	0.24	0.15	0	0	0	0
c233663_g1_4	C3H	0	0.74	0.11	0.07	0	0	0	0
c242957_g1_3	C3H	0	1.18	0.34	0.38	0	0	0	0
c216603_g1_5	C3H	0	2.42	0.16	0.09	0	0	0	0
c233229_g2_0	C3H	0	0.51	0.02	0.03	0	0	0	0
c242068_g1_0	C3H	0	1.27	0.27	0.22	0	0	0	0
c233063_g1_3	C3H	0	0.66	0.12	0.20	0	0	0	0
c226142_g2_3	C3H	0	0.50	0.10	0.10	0	0	0	0
c228857_g1_0	C3H	0	0.64	0.10	0.13	0	0	0	0
c242068_g2_1	C3H	0	0.71	0.30	0.16	0	0	0	0
c235657_g2_5	C3H	0	0.65	0.18	0.14	0	0	0	0
c223826_g1_3	C3H	0	0.56	0.10	0.25	0	0	0	0
c246101_g1_1	C3H	0	1.00	0.30	0.32	0	0	0	0
c232614_g1_0	C3H	0	0.82	0.11	0.11	0	0	0	0
c221111_g1_1	CCAAT	0	1.26	0.35	0.11	0	0	0	0
c226372_g1_4	CCAAT	0	1.64	0.15	0.29	0	0	0	0
c229435_g1_5	CCAAT	0	1.57	0.32	0.36	0	0	0	0
c238484_g4_2	CCAAT	0	2.13	0.40	0.64	0	0	0	0
c238484_g1_0	CCAAT	0	9.71	2.47	2.39	0	0	0	0
c238484_g2_1	CCAAT	0	2.18	0.45	0.46	0	0	0	0
c247108_g1_0	CSD	0	0.94	0.25	0.19	0	0	0	0
c220446_g1_0	CSD	0	0.69	0.34	0.16	0	0	0	0

c222090_g1_3	CSD	0	3.06	1.20	0.85	0	0	0	0
c223047_g1_5	E2F-DP	0	12.24	0.10	0.10	0	0	0	0
c249655_g1_1	EIL	0	5.74	0.86	0.60	0	0	0	0
c249655_g4_1	EIL	0	0.77	0.20	0.07	0	0	0	0
c244219_g1_1	FAR1	0	0.36	0.10	0.09	0	0	0	0
c217032_g1_0	G2-like	0	1.48	0.25	0.27	0	0	0	0
c225490_g1_4	G2-like	0	0.61	0.29	0.19	0	0	0	0
c220885_g1_0	GeBP	0	0.38	0.16	0.14	0	0	0	0
c240076_g1_4	GeBP	0	0.94	0.20	0.16	0	0	0	0
c235629_g1_3	GNAT	0	1.24	0.45	0.31	0	0	0	0
c220683_g1_1	GRAS	0	0.47	0.10	0.19	0	0	0	0
c229318_g1_0	GRAS	0	0.47	0.11	0.09	0	0	0	0
c226875_g1_0	GRAS	0	0.89	0.09	0.10	0	0	0	0
c239651_g1_3	GRAS	0	0.66	0.15	0.23	0	0	0	0
c232484_g1_4	GRAS	0	0.72	0.15	0.08	0	0	0	0
c241060_g1_1	GRAS	0	0.81	0.23	0.26	0	0	0	0
c244466_g1_3	GRAS	0	0.70	0.24	0.33	0	0	0	0
c243639_g1_1	HB	0	1.11	0.21	0.29	0	0	0	0
c228070_g1_4	HB	0	1.66	0.12	0.35	0	0	0	0
c243639_g2_2	HB	0	1.52	0.54	0.41	0	0	0	0
c237395_g1_0	HB	0	0.51	0.22	0.20	0	0	0	0
c231433_g1_2	HMG	0	0.92	0.33	0.45	0	0	0	0
c227315_g1_5	HMG	0	0.83	0.14	0.08	0	0	0	0
c242475_g1_0	HMG	0	10.06	2.00	2.38	0	0	0	0

c237971_g2_2	IWS1	0	0.97	0.47	0.42	0	0	0	0
c238539_g1_1	Jumonji	0	0.64	0.24	0.19	0	0	0	0
c224172_g1_3	LIM	0	0.45	0.13	0.23	0	0	0	0
c227796_g1_3	LOB	0	0.68	0.47	0.24	0	0	0	0
c237477_g1_3	LOB	0	2.71	1.17	0.71	0	0	0	0
c241569_g1_0	LOB	0	1.83	0.79	0.72	0	0	0	0
c241569_g1_1	LOB	0	1.83	0.79	0.72	0	0	0	0
c215046_g1_3	MADS	0	2.55	0.64	0.49	0	0	0	0
c242222_g1_0	MADS	0	2.39	0.37	0.51	0	0	0	0
c232118_g1_1	MADS	0	1.30	0.32	0.31	0	0	0	0
c230653_g1_3	MADS	0	0.89	0.22	0.38	0	0	0	0
c241438_g3_0	MADS	0	1.44	0.04	0.41	0	0	0	0
c234936_g2_3	MADS	0	3.11	0.82	1.05	0	0	0	0
c228961_g1_0	MBF1	0	1.60	0.28	0.51	0	0	0	0
c236739_g1_3	MYB	0	0.39	0.04	0.07	0	0	0	0
c225566_g1_1	MYB	0	0.71	0.05	0.11	0	0	0	0
c227734_g1_3	MYB	0	0.63	0.03	0.08	0	0	0	0
c229877_g1_2	MYB	0	1.02	0.16	0.19	0	0	0	0
c220104_g1_4	MYB	0	1.38	0.16	0.41	0	0	0	0
c230735_g1_2	MYB	0	0.78	0.22	0.21	0	0	0	0
c238455_g1_3	MYB	0	1.28	0.29	0.33	0	0	0	0
c235342_g1_2	MYB	0	0.34	0.03	0.08	0	0	0	0
c238031_g1_3	MYB	0	2.28	0.62	0.68	0	0	0	0
c246572_g2_1	MYB	0	1.28	0.43	0.54	0	0	0	0

c247371_g2_0	MYB	0	3.21	0.84	0.95	0	0	0	0
c237630_g1_3	NAC	0	1.00	0.36	0.16	0	0	0	0
c227932_g1_5	NAC	0	3.99	0.41	1.46	0	0	0	0
c231112_g1_3	NAC	0	0.69	0.07	0.11	0	0	0	0
c237489_g1_0	NAC	0	0.48	0.14	0.08	0	0	0	0
c239421_g1_2	NAC	0	1.72	0.20	0.84	0	0	0	0
c239609_g1_2	NAC	0	1.31	0.16	0.27	0	0	0	0
c239609_g2_2	NAC	0	0.70	0.17	0.06	0	0	0	0
c236429_g1_1	NAC	0	4.18	1.14	1.19	0	0	0	0
c42911_g1_5	NAC	0	0.47	0.04	0.06	0	0	0	0
c227072_g1_1	OFP	0.02	1.55	0.77	0.57	0	0	0	0
c210617_g1_3	Orphans	0	5.62	1.24	1.39	0	0	0	0
c237847_g2_1	Orphans	0	1.11	0.14	0.33	0	0	0	0
c227702_g1_1	Orphans	0	1.63	0.91	0.86	0	0	0	0
c232507_g1_4	Orphans	0	0.68	0.47	0.22	0	0	0	0
c219862_g1_2	Orphans	0	0.65	0.09	0.12	0	0	0	0
c236520_g1_1	Orphans	0	1.06	0.16	0.18	0	0	0	0
c247002_g2_2	Orphans	0	2.29	0.72	0.63	0	0	0	0
c225658_g1_1	PHD	0	0.31	0.08	0.17	0	0	0	0
c245257_g1_2	PHD	0	1.72	0.65	0.69	0	0	0	0
c242834_g2_3	PLATZ	0	1.29	0.16	0.53	0	0	0	0
c228713_g1_2	S1Fa-like	0	0.75	0.14	0.27	0	0	0	0
c242876_g1_1	SBP	0	2.14	0.45	0.53	0	0	0	0
c247644_g1_4	SBP	0	0.88	0.30	0.34	0	0	0	0

c199382_g1_3	SBP	0	0.46	0.06	0.05	0	0	0	0
c223399_g1_0	SNF2	0	0.41	0.19	0.10	0	0	0	0
c232171_g1_0	SWI/SNF-BAF60b	0	0.57	0.10	0.10	0	0	0	0
c220094_g1_3	SWI/SNF-BAF60b	0	1.77	0.40	0.40	0	0	0	0
c234026_g2_2	TAZ	0	0.41	0.02	0.05	0	0	0	0
c224843_g1_2	TAZ	0	0.36	0.14	0.11	0	0	0	0
c208831_g1_2	Tify	0	0.28	0.13	0.07	0	0	0	0
c229304_g1_2	Tify	0	0.82	0.19	0.16	0	0	0	0
c229304_g2_1	Tify	0	0.95	0.21	0.23	0	0	0	0
c233203_g1_4	TRAF	0	0.72	0.26	0.43	0	0	0	0
c246986_g2_2	TRAF	0	0.91	0.19	0.25	0	0	0	0
c238258_g1_1	Trihelix	0	0.54	0.17	0.25	0	0	0	0
c233328_g1_1	TUB	0	0.67	0.22	0.20	0	0	0	0
c223008_g1_0	TUB	0	0.41	0.04	0.11	0	0	0	0
c233958_g1_5	ULT	0	1.08	0.24	0.33	0	0	0	0
c219780_g1_4	WRKY	0	1.00	0.45	0.28	0	0	0	0
c225666_g1_3	WRKY	0	0.66	0.12	0.19	0	0	0	0
c235441_g1_2	WRKY	0	0.67	0.15	0.26	0	0	0	0
c223200_g2_5	WRKY	0	0.48	0.08	0.08	0	0	0	0
c230628_g1_0	WRKY	0	0.85	0.66	0.34	0	0	0	0
c237003_g1_1	WRKY	0	0.82	0.18	0.13	0	0	0	0
c221419_g1_4	WRKY	0	1.65	0.32	0.26	0	0	0	0
c238104_g1_3	WRKY	0	2.23	0.60	0.69	0	0	0	0
c210018_g2_3	WRKY	0	0.42	0.09	0.06	0	0	0	0

c223200_g1_5	WRKY	0	0.38	0.07	0.10	0	0	0	0
c236115_g1_1	WRKY	0	0.80	0.20	0.22	0	0	0	0
c239959_g1_3	WRKY	0	0.74	0.15	0.18	0	0	0	0
c239295_g1_1	zf-HD	0	1.61	0.33	0.36	0	0	0	0

Table S2. The expression of specific transcription factors in apomictic *Boehmeria tricuspis*.

Gene ID	Family	FPKM value							
		AI	AII	AIII	AIV	SI	SII	SIII	SIV
c239459_g1_2	AP2-EREBP	0	0	0	0	0	0.45	0.42	0
c244141_g1_2	AP2-EREBP	0	0	0	0	0	0.43	0.66	0
c229471_g1_1	AP2-EREBP	0	0	0	0	0	0.56	0.71	0
c233462_g2_5	bZIP	0	0	0	0	0	0.90	0.83	0
c80750_g1_0	bZIP	0	0	0	0	0	0.32	0.50	0
c193850_g1_5	C2H2	0	0	0	0	0	0.53	0.54	0
c218612_g1_4	C3H	0	0	0	0	0	1.01	1.81	0
c259192_g1_5	HMG	0	0	0	0	0.03	0.19	0.17	0.38
c237804_g1_3	LOB	0	0	0	0	0	0.69	0.90	0
c246445_g2_2	MADS	0	0	0	0	0	0	0.26	1.23
c81669_g1_5	MYB	0	0	0	0	0	0.74	0.56	0
c230428_g1_1	NAC	0	0	0	0	0	0.60	0.62	0
c237011_g5_3	NAC	0	0	0	0	0	0.61	1.01	0
c221339_g1_1	Orphans	0	0	0	0	3.17	4.94	0.41	0
c313408_g1_1	Orphans	0	0	0	0	2.84	0	0	0
c205490_g1_0	TAZ	0	0	0	0	0	0.15	0.55	0

c239459_g1_2	AP2-EREBP	0	0	0	0	0	0.45	0.42	0
c244141_g1_2	AP2-EREBP	0	0	0	0	0	0.43	0.66	0
c229471_g1_1	AP2-EREBP	0	0	0	0	0	0.56	0.71	0
c233462_g2_5	bZIP	0	0	0	0	0	0.90	0.83	0

Table S3. The expression of specific transcription factors in sexual *Boehmeria tricuspis*.

Gene ID	Swiss-Prot Description	FPKM value							
		AI	AII	AIII	AIV	SI	SII	SIII	SIV
c240291_g1	argonaute 5	0	4.19	1.75	0.47	0	0	0	0
c245552_g1	argonaute 5	0	3.48	1.06	0.98	0	0	0	0
c224550_g1	argonaute 4A	0	4.85	1.51	1.01	0	0	0	0
c239761_g1	argonaute 4	0	2.03	0.68	0.81	0	0	0	0
c233608_g1	argonaute 4	0	1.92	0.46	0.45	0	0	0	0
c232753_g1	argonaute 4	0	2.49	0.52	0.67	0	0	0	0
c250672_g1	argonaute 1B	0	6.68	1.51	1.4	0	0	0	0
c227166_g1	argonaute 1	0	2.74	0.56	1.04	0	0	0	0

Table S4. The expression of specific *AGO* transcripts in apomictic *Boehmeria tricuspis*.

Gene ID	Primer sequence	Gene ID	Primer sequence
c154540_g1	CACACACAGACACAAGGACG	c213177_g1	AGGGAACACATGGGGCATAA
	CTACACCATCAGCACCTCCA		GCAAGAGGAAGCGAGAAAGG
c225929_g1	AAGTGCGGTGACCATTGTTC	c231022_g1	TCCCCTACAGCTTGCTTGAA
	GGGGCATTTGAACAAGCAGA		CTCAGCTTTCGTCTGATGGC
c231538_g1	CTCTTTGCGTCATCCTCAGC	c235751_g1	CGGTTTTGAAGCCAGTGACA

	ATGTTGGTTTTCATGCGGCT		CACCACCTTTTCCTCCTCCT
c242116_g1	TCCCCTCTTCTCCCAAACAC	c244577_g1	CGGACATGGAGGCTTTCAAG
	GGAGGAGGAAAGCGGATGTA		CGATCCTGCAACGTTTAGGG
c246323_g1	GGGAAGGAAGGGAACATGGA	c247631_g5	TCCATCCATATCAGGTCCGC
	AGCAGATGGAGCAGGAATGT		TCTTCTGGGAGTGGGACCTA
c250402_g3	CTCGGGCTTTTCGTGATGAC	c251767_g2	GGCCCAACCCAAATGCTAAA
	TGAAGCAAACAAGGACGTGG		TCATGATCTTTGTGGCAGCG
c252400_g4	ACATGTCGTTTCGGTTGCAA	c252858_g2	TGGATAAGCGTCGGTCACTT
	TCTCTGCCTCATAACCCCAA		TGCCTCGGTAATGGCGATAT
EF1 α	ATCAAGCCCACTAAGCCCTT		
	TTCTCTCTGGCAGTGACCTC		

Table S5. List of primers sequences used for real time PCR analysis.
